# Combined central and peripheral demyelination: a case report

**DOI:** 10.25122/jml-2022-0010

**Published:** 2022-11

**Authors:** Elena Costru-Tasnic, Elena Manole, Vitalie Lisnic

**Affiliations:** 1 Neurology Department No. 1, Nicolae Testemițanu State University of Medicine and Pharmacy, Chisinau, Republic of Moldova

**Keywords:** chronic inflammatory demyelinating polyneuropathy, multiple sclerosis, combined central and peripheral demyelination, immunotherapy, AIDP – acute inflammatory demyelinating polyneuropathy, CCPD – combined central and peripheral demyelination, CIDP – chronic inflammatory demyelinating polyneuropathy, CNS – central nervous system, CSF – cerebrospinal fluid, IVIg – intravenous immunoglobulin, LE – lower extremity, MRC – Medical Research Council, MRI – magnetic resonance imaging, MS – multiple sclerosis, NCS – nerve conduction studies, PLEX – plasma exchange, PNS – peripheral nervous

## Abstract

Overlapping central nervous system (CNS) and peripheral nervous system (PNS) demyelination is a rare clinical entity, more frequently seen in patients with chronic inflammatory demyelinating polyneuropathy (CIDP) and multiple sclerosis (MS). This case report showcases a patient with atypical CIDP and CNS demyelination lesions. Demographic data, disease course, treatment responsiveness, neurological examination, laboratory tests, nerve conduction studies (NCS), and brain and spinal cord MRI were registered. The case highlights the difficulty of diagnosis establishment and treatment selection, given the atypical course of the disease and limited answers to the indicated therapies. The data from our report suggest that specific and widely available immunological targets are necessary for diagnosing combined central and peripheral demyelination cases appropriately. The association of different immunotherapeutic agents may be necessary to induce and maintain disease remission.

## INTRODUCTION

Chronic inflammatory demyelinating polyneuropathy (CIDP) is an acquired, immune-mediated demyelinating neuropathy with a reported prevalence of 0.7–10.3 cases per 100,000 people, characterized by a relapsing-remitting or progressive course, glucocorticoid responsiveness, and electrodiagnostic or pathologic features of demyelination [1].

Clinically, in its typical form, CIDP progresses over at least two months with more accentuated weakness than sensory symptoms, symmetric distribution in all limbs, proximally and distally muscle involvement, widespread reduction or absence of deep tendon reflexes, and gait ataxia [1]. Ancillary tests include cerebrospinal fluid (CSF) analysis with increased protein without pleocytosis, nerve conduction studies (NCS) – demyelinating neuropathy, and nerve biopsy showing segmental demyelination with or without inflammation [1]. Several noncontrolled studies have suggested concomitant inflammatory central nervous system (CNS) demyelination similar to multiple sclerosis [2]. Cortese A et al. revealed manifestations of CNS involvement in up to one-third of CIDP cases [3].

Coincident multiple sclerosis (MS) and inflammatory polyneuropathy were described for the first time in 1979 by Forrester C and Lascelles RG [4]. In 1987 Thomas PK et al. described 6 additional cases of CIDP with CNS demyelination evidenced by magnetic resonance imaging (MRI) [5].

Recently, more reports emphasized that MS does not have a strictly CNS distribution, with the peripheral nervous system (PNS) being affected in various degrees in MS patients [6]. In a study performed on 50 consecutive MS patients, electrophysiologic abnormalities of the peripheral nerves were observed in 28% of cases, with concomitant clinical signs in 12% [7].

Japanese, Chinese, and German researchers have recently suggested the existence of a condition termed combined central and peripheral demyelination (CCPD) that comprises the cases in which both CNS and PNS demyelination is registered, more frequently resembling MS and CIDP in the same patient [8–10]. The diagnosis and treatment remain challenging in these situations, based on clinical judgment and experience [3].

In this paper, we present a case report of a young patient diagnosed with CIDP and CNS demyelination as part of the combined central and peripheral demyelination syndrome.

## CASE REPORT

A 27-year-old man, previously healthy, presented his first neurological symptoms in October 2018, particularly – acute onset of short lasting (4 days), self-limited episode of numbness in both hands, and heavy legs sensation. In February 2019, the patient was admitted to the neurological department, given the acute onset of distal weakness in lower limbs, weakness in hands, and diplopia with the progression of weakness to tetraplegia over the next 4 days. Non-contrast brain MRI noticed several lesions suggestive of demyelination, and a presumptive diagnosis of multiple sclerosis was established. Pulse therapy (methylprednisolone 1000 mg/day, 5 days) did not improve the clinical state, and plasma exchange (PLEX) administered over 4 sessions had no significant impact on the neurological symptoms.

The patient remained tetraplegic with pain along the spine and limbs. The general examination, family, and past medical history were unremarkable.

The neurological exam revealed both sides of facial palsy, flaccid tetraplegia (MRC 0/5 points) with diffuse hypo-/areflexia; decreased superficial sensibility in length-dependent pattern ("socks and gloves"), positive Lasegue (70°) and Neri signs.

The laboratory evaluation exam: complete blood count – mild neutrophilic leucocytosis (13.3), ↑ erythrocyte sedimentation rate (24 mm/h), ↑ C-reactive protein (24 IU/ml); urine analysis – cloudy urine, bacteriuria (+), mild leukocyturia (2–4 cells/visual field); blood chemistry – mild elevation of liver and pancreas enzymes; chest X-ray – unremarkable; CSF analysis: transparent; increased proteins (2.04 g/l); normal glucose; mildly increased cells 38 v/f (neutrophils 85%, lymphocytes 15%; 18–20 v/f unmodified erythrocytes); anti-aquaporin 4 IgG – negative; oligoclonal band – equivocal positive result (1 band detected).

**Figure 1:**
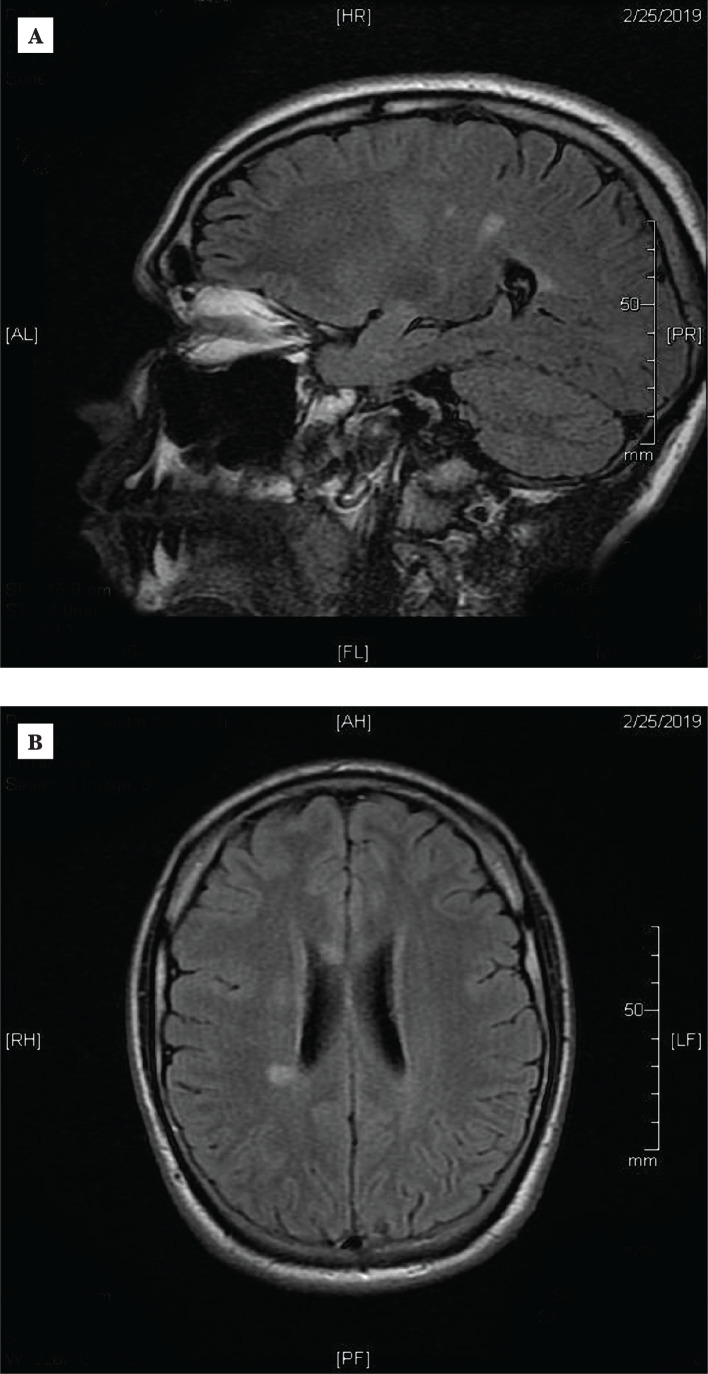
Axial fluid-attenuated inversion recovery (FLAIR) MRI sections revealing hyperintense contrast-enhancing lesions in the corpus callosum and periventricular regions (A and B).

**Figure 2:**
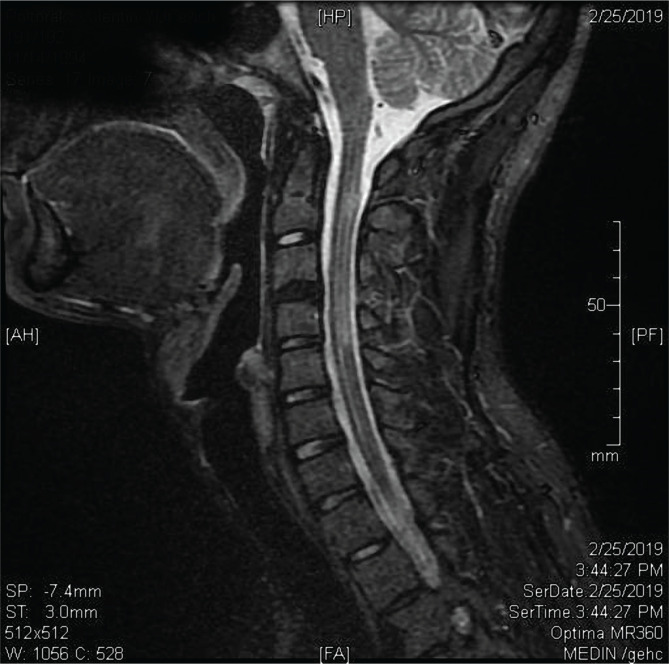
Sagittal T2-weighted MRI sequence of the cervical spinal cord revealing nonhomogeneous cervical spinal cord with a focal area of centro-posterior myelopathy (C1-C2: 3×4×15 mm), non-enhancing (white arrows).

Diagnostic procedures:

- Nerve conduction studies – demyelinating polyneuropathy (Table 1);
- Brain MRI – multiple lesions in the projection of corpus callosum suggestive of demyelination – foci of demyelination with gadolinium enhancement (Figure 1);
- Cervical MRI – nonhomogeneous cervical spinal cord with a focal area of centro-posterior myelopathy (C1-C2: 3×4×15 mm), non-enhancing (Figure 2).

**Table 1:** A summary of nerve conduction studies performed over 3 years (2018–2021) demonstrating demyelinating polyneuropathy.

Motor NCS														
Nerve	Site		Latency (ms)				Amplitude (mV)				Conduction velocity (m/s)			
			Year of examination											
			2018	2019	2020	2021	2018	2019	2020	2021	2018	2019	2020	2021
Median	Right	Wrist	-	6.9	9.5	5.3	-	0.1	2.1	4.3	-	-	-	-
		Elbow	-	17.35	20.2	11.7	-	0.08	0.7*	4.3	-	27.4	19.7	42
	Left	Wrist	-	13.8	10.5	4.8	-	0.3	0.4	6.5	-	-	-	-
		Elbow	-	25.81	21.3	11.4	-	0.26	0.4	6.1	-	18.8	19.4	31.8
Ulnar	Right	Wrist	-	11.2	NR	4.1	-	0.3	NR	4.5	-	-	-	-
		Elbow	-	23.56	NR	11.2	-	0.28	NR	3.0	-	26.7	NR	25.4
		Above elbow	-	-	NR	13.1	-	-	NR	5.8	-	-	48.8	54.1
	Left	Wrist	-	11.4	6.5	4.1	-	-	0.5	4.8	-	-	-	-
		Elbow	-	23.27	18.4	11.5	-	0.3	NR	5.7	-	24.2	15.2	24.3
		Above elbow	-	-	21.5	13.4	-	-	NR	8.5	-	-	32.3	51.3
Peroneal	Right	Ankle	3.8	4.1	NR	NR	9.7	1.2	NR	NR	-	-	-	-
		Fibular head	10.2	11.23	NR	NR	9.2	1.14	NR	NR	50.0	44.2	NR	NR
		Popliteal	12.0	12,6	NR	NR	9.0	1.11	NR	NR	56.6	47.5	NR	NR
	Left	Ankle	6.8	8.1	NR	NR	7.8	0.3	NR	NR	-	-	-	-
		Fibular head	13.4	16.02	NR	NR	7.5	0.3	NR	NR	45.1	42.3	NR	NR
		Popliteal	15.6	18.2	NR	NR	7.6	0.3	NR	NR	46.3	47.0	NR	NR
Tibial	Right	Ankle	3.5	4.6	NR	NR	7.2	0.4	NR	NR	-	-	-	-
		Popliteal	12.3	12.96	NR	NR	5.2	0.4	NR	NR	45.5	44.6	NR	NR
	Left	Ankle	3.2	-	NR	NR	8.2	1.2	NR	NR	-	-	-	-
		Popliteal	11.6	8.06	NR	NR	5.7	1.16	NR	NR	47.9	NR	NR	NR
Sensory NCS														
Nerve			**Site**			**Peak Latency (ms)**			**Amplitude (µV)**			**Conduction velocity (m/s)**		
						**Year of examination**								
						**2018**	**2020**	**2021**	**2018**	**2020**	**2021**	**2018**	**2020**	**2021**
Superficial			Right			1.3	15.5	2.5	9.4	1.6	1.3	53.8	6.5	40.0
Peroneal			Left			2.0	18.1	1.8	8.8	2.8	1.1	54.3	5.5	57.1
Late responses														
			**Latency (ms)**											
			**2018**						**2021**					
R Tibial F-wave			46.6						53.7					
L Tibial F-wave			47.9						-					

NCS – nerve conduction studies; NR – not recorded; * – Conduction block.

The treatment included PLEX (6 sessions) and IVIg (2 g/day – 5 days) with subsequent neurological improvement, namely the partial recovery of muscle strength (from 0 to 1–2 points). The patient started an in-hospital rehabilitation course, continued by an at-home recovery program and continuous neurological state amelioration.

Six months later, the patient's state worsens with the occurrence of weakness in the hands, followed by weakness in the legs on the second day. The patient developed tetraplegia again in one week. The neurological exam showed flaccid deep tetraparesis of the upper limb (UE), with 2/5 points for British Medical Research Council scale (BMRC) proximally and 1/5 points distally. In addition, the examination of the lower extremity (LE) revealed 2/5 points for MRC proximally and 0–1/5 points distally. Other manifestations included hands muscles hypotrophy, a slight decrease of superficial sensibility in length-dependent pattern ("socks"), and a positive Lasegue (50–60°) sign. Repeated CSF analysis showed increased proteins (2.44 g/l), normal glucose, and cells 1 v/f. On NCS – sensitive and motor myelinopathy (polyneuropathy) were registered (Table 1).

**Figure 3:**
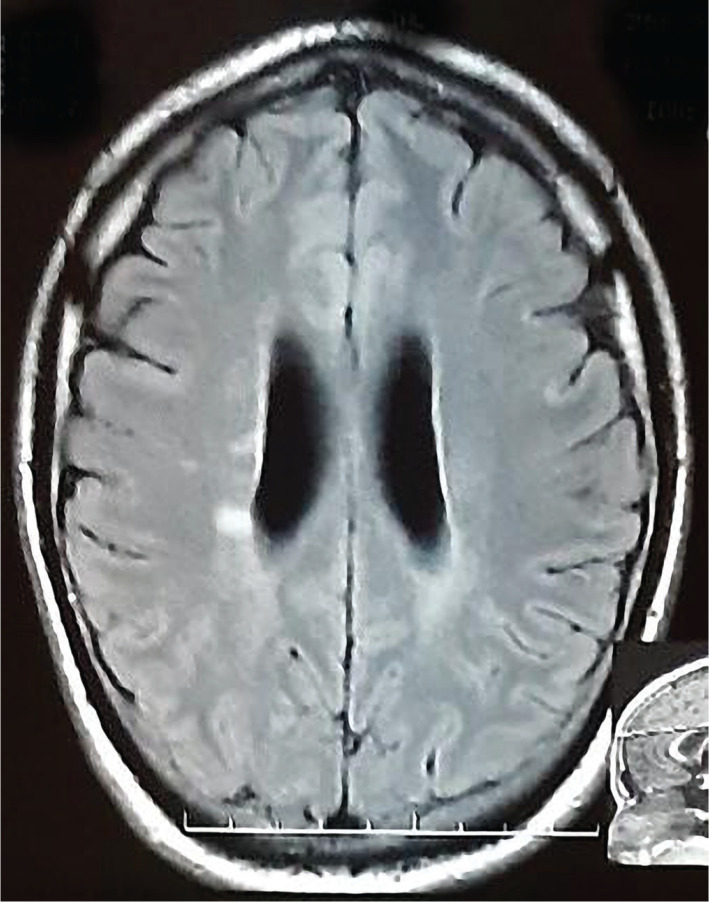
Axial fluid-attenuated inversion recovery (FLAIR) MRI section revealing an acute hyperintense contrast-enhancing lesion in the right periventricular region.

Brain MRI revealed multiple lesions, with an acute one in the right periventricular region (Figure 3).

The treatment approach included 6 PLEX sessions, Dexamethasone 16 mg/day for 3 days, followed by Prednisolone 60 mg/day. We noticed a positive evolution starting from day 5 of treatment (apparition of movements and mild increase of muscle strength from 0 to 1–2 points). At discharge (2 weeks later), the patient was able to sit unassisted and use his hand to manage daily living activities.

### Outcome and Follow-up

At one-month follow-up, the tetraplegia regressed to moderate tetraparesis, with distal muscles being more affected. Azathioprine 2 mg/kg/day was started, along with Prednisolone 1 mg/kg/day. After 3 months, Prednisolone was tapered, and 6 months later, he was Prednisolone-free. The clinical state was with continuous improvement. The patient was able to walk with unilateral assistance (walking crutch), despite the persistence of moderate distal paresis in LE. Our final diagnosis was atypical chronic inflammatory demyelinating polyradiculoneuropathy and CNS demyelination as part of the combined central and peripheral demyelination syndrome. The patient did not have any relapse of the disease for the last 2 years of follow-up.

## DISCUSSION

There are no consistent data to establish the incidence of simultaneous CNS demyelination in CIDP patients. An earlier study [7] revealed a 12.72% (7/55) incidence of CNS demyelination in CIDP-confirmed patients. These patients were younger but with worse clinical outcomes and required more aggressive treatment for relapse prevention. In comparison, in the Japanese study by Pineda et al. (2007), eighteen consecutive patients with probable CIDP were analyzed (electrophysiological and MRI investigations) to identify CNS lesions, and approximately one-third (5/18) presented subclinical CNS involvement, with male predominance, milder disease, and a favorable response to immunotherapies [11]. However, a more recent retrospective, observational Italian study (Cortese et al., 2016), including thirty-one CCPD patients (selected from 276 patients with idiopathic inflammatory diseases of the CNS or PNS), suggested that CCPD has heterogeneous features, primary peripheral nervous system or central nervous system involvement, poor response to treatments, and a generally worse outcome than separate entities affecting PNS or CNS apart [3], similarly to our earlier findings.

More particularly, the clinical presentation in most CCPD patients was with lower limb sensory-motor impairment and sphincter dysfunction, suggestive of CNS lesions. In 11 patients, PNS damage was the primary damage, and four subjects presented with pseudo-Guillain-Barré syndrome [3]. In their study, Cortese et al. noted that one-third of the patients had a monophasic disease course, and the other patients (68%) presented a progression of the disease: either a relapse, with subacute onset of new symptoms (13 cases, 42%), or a steady chronic progression (8 cases, 26%).

Similarly, in typical forms, CIDP patients present a subacute progression over at least two months [1]. Clinically, in CIDP, weakness is more prominent than sensory symptoms, with symmetric involvement of arms and legs, involvement of the proximal and distal muscles, widespread reduction or absence of deep tendon reflexes, and gait ataxia secondary to large fiber sensory loss [1]. Cortese et al. found lower limb weakness in 27 patients (87%), with distal predominance in five. The tone of the four limbs was increased in 11 patients, decreased in six, and mixed (increased in the upper limbs and decreased in the lower limbs) in two. Deep tendon reflexes were more often increased in the upper limbs (48% of the cases) and reduced or abolished in the lower limbs (52%).

Ancillary tests show increased CSF proteins without pleocytosis in typical CIDP, evidence of demyelinating neuropathy on NCS, and segmental demyelination with or without inflammation on nerve biopsy [1]. The CCPD patients have slightly different results, given the concomitant CNS damage, but have multiple similarities to CIDP data, like elevated total proteins, intrathecal IgG production, demyelination on NCS fulfilling the European Federation of Neurological Societies and the Peripheral Nerve Society (EFNS/PNS) electrodiagnostic criteria for CIDP [3].

Our case showed a patient with atypical CIDP and concomitant demyelination of the CNS. Misdiagnosis is common and reported in up to 50% of patients referred with a CIDP diagnosis, mainly in those with an atypical presentation [12]. One of the first challenges in the presented case was to differentiate between acute inflammatory demyelinating polyneuropathy (AIDP) and CIDP diagnosis. Up to 16% of patients with CIDP may demonstrate acute-onset CIDP, characterized by a rapidly progressive onset within 8 weeks [13], making difficult an early, correct diagnosis and leading to treatment failure and/or delay.

The difficulty of CIDP management also comes from the heterogeneous manifestations of the disease, with almost 40–50% of cases evolving in atypical variants [1]. Atypical CIDP may be divided, based on clinical presentation, into asymmetric, focal, distal, pure motor, and pure sensory variants [12].

Therefore, our patient had sub-acute onset and fast evolution of symptoms, bilateral facial nerve involvement, monophasic course (at first admission to our clinic), and a good answer to PLEX and IVIg, suggesting AIDP. Consecutively, after a new relapse, the CIDP diagnosis was established based on the timeframe and disease evolution, NCS modifications (demyelinating polyneuropathy with secondary axonal loss meeting the European Federation of Neurology/Peripheral Nerve Society guidelines for the diagnosis of definite CIDP [1]), and CSF changes (albumin-cytological dissociation). Given the slight asymmetric pattern of weakness which evolved in time, the presence of cranial neuropathies, and the transient response to intravenous immunoglobulin, plasma exchange, alongside a good response to corticosteroids and Azathioprine, we supposed a possible atypical form of CIDP, from the paranodal/nodal antibody positive (PNAb+) neuropathies group [11, 14]. However, the confirmatory analysis could not be performed.

The clinical attack (first episode of numbness 4 months prior to admission), MRI brain and spinal cord lesions, and the equivocal oligoclonal bans in CFS allowed for establishing the presence of CNS demyelination in our patient. The follow-up MRIs showed new lesions. However, their distribution could not explain the patient's symptoms, being attributed more probably to CIDP.

Available immunotherapies for patients with CCPD include steroids, IVIg, and plasmapheresis [15]. Recently, Rituximab has shown good results in patients with CCPD [3, 11]. Our last patient had a transitory response to plasmapheresis and IVIg, and switching to corticosteroids and immunosuppressive therapy (Azathioprine) improved his clinical state momentarily and prevented new relapses up to this moment. An alternative treatment, in this case, could have been immunomodulation therapy with Rituximab.

## CONCLUSION

Overlapping CNS and PNS demyelination is a challenging clinical entity, both for diagnosis and treatment. The presented case highlights the necessity of specific and widely available immunological targets for appropriate diagnosis. A combination of different immunotherapeutic agents may be necessary to induce and maintain disease remission.
